# Regulatory Mechanism of ITGBL1 in the Metastasis of Colorectal Cancer

**DOI:** 10.3389/fonc.2020.00259

**Published:** 2020-03-10

**Authors:** Lu Qi, Fuyao Song, Yanqing Ding

**Affiliations:** ^1^Department of Pathology, Nanfang Hospital, Southern Medical University, Guangzhou, China; ^2^Department of Pathology, School of Basic Medical Sciences, Southern Medical University, Guangzhou, China; ^3^Guangdong Provincial Key Laboratory of Molecular Oncologic Pathology, Guangzhou, China

**Keywords:** colorectal cancer, integrin, beta-like 1 (ITGBL1), Wnt signaling pathway, tumor microenvironment

## Abstract

Integrin, beta-like 1 (ITGBL1) protein is located in the extracellular matrix (ECM) and involved in the development and metastasis of many tumors. However, the regulatory mechanism of ITGBL1 in colorectal cancer (CRC) remains unclear. This study was to analyze the expression profile of CRC and to identify the expression change of *ITGBL1* gene at different stages of CRC. Survival analysis showed that *ITGBL1* was related to the metastasis of CRC, and CRC patients with a high expression of *ITGBL1* had earlier metastasis. Gene Set Enrichment Analysis (GSEA) indicated the relationship between *ITGBL1* expression and molecular events of CRC. The results indicated that a high expression of *ITGBL1* was linked to Wnt signaling pathway, cell polarity, and tissue development, while a low expression of *ITGBL1* was related to cellular respiration, electron transfer chain, and oxidative phosphorylation. With the expression profiles from interstitial and parenchyma CRC tissues, a comparison was made to determine the difference between high/low expression of *ITGBL1* and Wnt signaling pathway, respectively, and further confirmed the close relation between *ITGBL1* and Wnt signaling pathway. To determine the relation, an interaction network of ITGBL1 and Wnt signaling proteins was constructed. It was found that β-catenin interacted with multiple extracellular Wnt signals and could bind to ITGBL1. As a result, the regulatory mechanism of ITGBL1 in CRC is related to extracellular Wnt signals and may affect extracellular Wnt signals via β-catenin.

## Introduction

Integrin, beta-like 1 (ITGBL1) is a β-integrin-related extracellular matrix (ECM) protein. Recently, studies on ITGBL1 have been increasing, and it was reported that *ITGBL1* could promote bone metastasis of breast cancer through transforming growth factor (TGF)-β signaling pathway (1). Studies showed that ITGBL1 could promote the invasion of ovarian cancer cell through Wnt/planar cell polarity (PCP) signaling and focal adhesion kinase (FAK)/Src pathway (2), and high expression of ITGBL1 was related to the poor prognosis and drug resistance of ovarian cancer (3). In gastric cancer, ITGBL1 was linked to epithelial–mesenchymal transition (EMT) phenotype and poor prognosis (4). Studies reported that hypermethylation of *ITGBL1* was correlated with poor prognosis of acute myeloid leukemia (5). Furthermore, studies also revealed that ITGBL1 could activate nuclear factor (NF)-κB signaling pathway and promote the EMT, invasion, and migration of prostatic cancer (6). The abovementioned studies revealed that ITGBL1 was associated with the invasion and metastasis of tumors. It was reported that *ITGBL1* was significantly upregulated in CRC, and its high expression was related to shortened survival of CRC patients. Additionally, knockdown of *ITGBL1* suppressed CRC cell proliferation, migration, and invasion (7). Another study showed that *ITGBL1* was associated with the overall survival rate (OSR) and relapse-free survival (RFS) of CRC patients, and subgroup validation demonstrated that a high *ITGBL1* expression was correlated with shorter RFS in stage II patients, which suggested that *ITGBL1* was a promising candidate biomarker for predicting the relapse of CRC (8). In the earlier study, a comparison of CRC expression data from normal to distant organ metastasis (normal, stage I, II, III, IV, liver, and lung metastasis) was made. There was screening of 39 genes with continuously increasing expression which contained *ITGBL1* (9). These studies showed that ITGBL1 played a vital role in the development of CRC. ITGBL1 was involved in the formation of tumor microenvironment, but the molecular mechanism of ITGBL1 in CRC remained unclear. Therefore, this study aims to analyze the molecular mechanism of ITGBL1 in CRC and determine the regulatory mechanism of ITGBL1 in the metastasis of CRC.

## Materials and Methods

### *ITGBL1* Expression Analysis in Colorectal Cancer

It was found previously that the expression of *ITGBL1* was continually increasing in CRC. In this study, CRC expression profiles GSE41258 (54 normal cases, 28 stage I cases, 50 stage II cases, 49 stage III cases, 58 stage IV cases, 47 liver metastasis cases, and 20 lung metastasis cases) from the Gene Expression Omnibus (GEO) database (10) which showed the expression of *ITGBL1* at different stages of CRC were analyzed. Additionally, we confirmed the expression of *ITGBL1* through expression profile GSE49355 and RNA-sequencing GSE50760. GSE49355 included 18 normal cases and 20 CRC cases with primary focus and 19 CRC cases with liver metastasis. GSE50760 included 18 normal cases, 18 CRC cases with primary focus, and 18 CRC cases with liver metastasis. *ITGBL1* gene expression in that data was obtained and divided into different groups according to tumor progression. The comparison of the difference in each group was performed by one-way ANOVA test, and *P*-value was calculated by Kruskal–Wallis test.

### Survival Analysis of *ITGBL1* in Colorectal Cancer

*ITGBL1* gene expression was continuously increasing in CRC, and many studies reported that ITGBL1 was associated with tumor metastasis. Therefore, a survival analysis of *ITGBL1* gene expression and CRC metastasis was conducted by using expression profile GSE28722 from the GEO database. GSE28722 included 125 CRC cases with data of survival times and metastasis. *ITGBL1* gene expression in those data was obtained and divided into the high *ITGBL1* expression group (*n* = 62) and the low *ITGBL1* expression group (*n* = 62) according to a median value (deleting median). Kaplan–Meier curve (11) was used to depict the survival curve of the two groups, and log rank test (12) was performed to analyze the statistical difference between the two groups with the *P*-value calculated. Considering the American Joint Committee on Cancer (AJCC) staging and the effect of patients' age (taking 60-year-olds as the dividing point) on metastasis, and Cox proportional-hazards regression was performed to further determine the effect of *ITGBL1* on metastasis.

### Molecular Mechanism Analysis of ITGBL1 in Colorectal Cancer

To clarify the molecular mechanism of ITGBL1 in CRC, an analysis of the dataset GSE39582 from the GEO database was carried out. GSE39582 included 566 CRC cases, which were divided into the high *ITGBL1* expression group (*n* = 283) and the low *ITGBL1* expression group (*n* = 283) according to the median value of *ITGBL1* expression (probe ID: 205422_s_at) based on the Gene Ontology (including biological process, molecular function, and cellular component) and signaling pathway (including KEGG pathway and REACTOME pathway). Enrichment analysis was performed on the high *ITGBL1* expression group and the low *ITGBL1* expression group by using Gene Set Enrichment Analysis (GSEA) [false discovery rate (FDR) < 25%, nominal *p* < 1%], with the version of gene set as V7.0 (13). ITGBL1 was mainly located in the ECM and related to the tumor microenvironment, so GSE35602 was used to screen the *ITGBL1*-related differentially expressed gene (DEG) in the parenchyma and interstitial of CRC. GSE35602 included 12 cases with CRC parenchymal tissue data and 12 cases with CRC interstitial tissue data. According to the median value of *ITGBL1* expression (probe ID: A_23_P408363), *ITGBL1* expression data from CRC parenchymal tissue and interstitial tissue were divided in the high *ITGBL1* expression group (*n* = 6) and the low *ITGBL1* expression group (*n* = 6), respectively. GEO2R (14) was used to screen DEG in the high *ITGBL1* expression group and the low *ITGBL1* expression group with the *P*-value limited < 0.01 and the fold change as 4. Based on the DEG, ITGBL1-related molecular events in parenchymal and interstitial tissues were examined to elicit the differences between them.

## Results

### *ITGBL1* Expression Change in Colorectal Cancer

**Figure 1:**
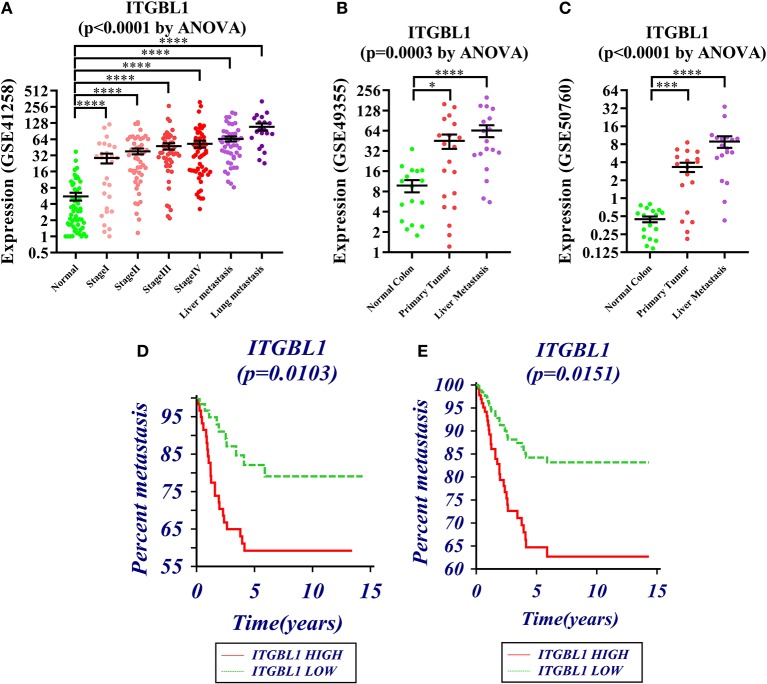
**(A–C)** Expression changes of *ITGBL1* in the development and metastasis of colorectal cancer (CRC) (*P < 0.05, **P < 0.01, ***P < 0.001, ****P < 0.0001). **(D)** Survival curve of *ITGBL1* and the metastasis time of CRC patients (Kaplan–Meier survival estimate and univariable survival analysis model). **(E)** Survival curve of *ITGBL1* and the metastasis time of CRC patients (Cox proportional hazards regression model and multivariate analysis model, excluding the influence of tumor stages and ages on metastasis time).

GSE41258, GSE49355, and GSE50760 showed the expression change of *ITGBL1* during the progression of CRC. The results showed a significant difference of *ITGBL1* expression at different stages of CRC (*P* < 0.0001, *P* = 0.0003, and *P* < 0.0001). *ITGBL1* expression was continuously increasing with the development of CRC (Figures 1A–C), suggesting that *ITGBL1* played an important role in the development and metastasis of CRC.

### Survival Analysis of *ITGBL1* in Colorectal Cancer

The analysis on the association between *ITGBL1* expression and the metastasis of CRC patients through dataset GSE28722 showed that *ITGBL1* expression was correlated with the metastasis of CRC (log rank *P* = 0.0103) [hazard ratio (HR) = 0.3924 (95% CI: 0.1982–0.7772)] (Figure 1D). *ITGBL1* expression was negatively related to the metastasis-free survival, and a high expression of *ITGBL1* could promote the metastasis of CRC. Cox proportional-hazards regression showed that *ITGBL1* was an independent factor of CRC metastasis (*P* = 0.0151). Patients in the high *ITGBL1* expression group had a higher risk of metastasis than that of the low *ITGBL1* expression group [HR = 2.5345 (95% CI: 1.2012–5.3477)] (Figure 1E).

### Molecular Mechanism of ITGBL1 in Colorectal Cancer

GSEA was performed on the high *ITGBL1* expression group and the low *ITGBL1* expression group based on the GSE39582 dataset. It was found that in the high *ITGBL1* expression group, top 20 of biological process in enrichment were mainly related to Wnt signaling pathway, cell polarity, tissue development, axon, and morphogenesis. Cellular component was mainly associated with cell matrix junction, cytoskeleton, and cell membrane. Molecular function was related to ECM binding, adhesion molecule binding, integrin binding, and FRIZZLED protein binding. Signaling pathway related to high *ITGBL1* expression was mainly linked to cytoskeleton, Wnt signaling pathway, pathway in cancer, and pathway related to tumor development. In the low *ITGBL1* expression group, top 20 of biological process in enrichment were mainly related to cellular respiration, electron transfer chain, and oxidative phosphorylation (see Supplementary Material). Those results demonstrated that *ITGBL1* was involved in various molecular events in the progression of CRC and was mainly related to cell adhesion. Enrichment analysis showed that Wnt signaling pathway in the high *ITGBL1* expression group occurred frequently in the biological process, which suggested that *ITGBL1* might be closely related to Wnt signaling pathway.

**Figure 2:**
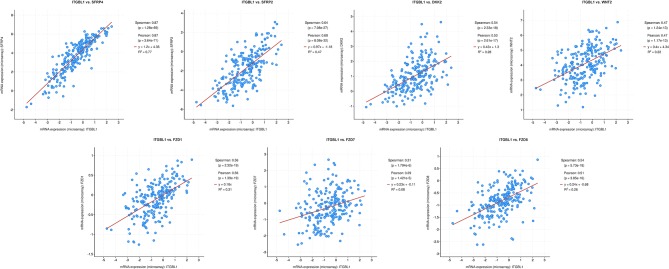
Co-expression relationship of *ITGBL1* and seven extracellular Wnt signals. Y axis in this figure represented mRNA relative expressions of these seven extracellular Wnt signals, and X axis represented mRNA relative expressions of ITGBL1. The red line indicated regression line.

**Figure 3:**
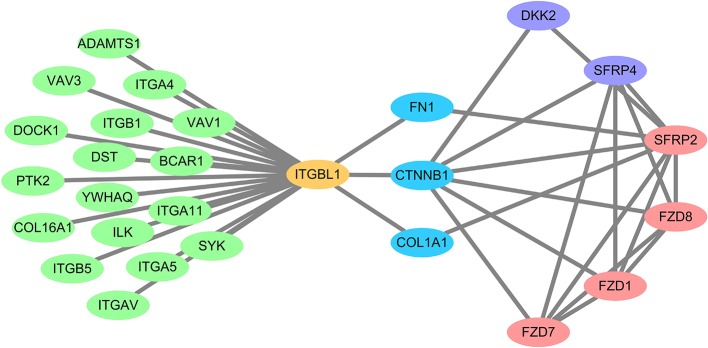
Interaction network of ITGBL1 and extracellular Wnt signal. The red nodes (SFRP2, FZD1, FZD7, FZD8) were extracellular Wnt signals that were upregulated in the stroma. The purple nodes (SFRP4, DKK2) were extracellular Wnt signals that were upregulated in both the stroma and parenchyma. The blue nodes (FN1, CTNNB1, COL1A1) were all ITGBL1 binding proteins, and they also bound to extracellular Wnt signal. The green nodes were ITGBL1 binding proteins. The yellow node was the ITGBL1 protein.

DEG screening was performed on CRC parenchymal tissue and interstitial tissue based on the dataset GSE35602. In parenchymal tissue, resulting in 137 upregulated genes (*SERP4* had the highest significant difference) and 17 downregulated genes in the high *ITGBL1* expression group. In interstitial tissue, there were 343 upregulated genes (*SERP2* and *SERP4* had the highest significant difference) and 38 downregulated genes in the high *ITGBL1* expression group. GSEA was used to analyze 12 cases with CRC interstitial tissue data, and the results were similar to GSE39582 analysis. This further confirmed the accuracy of the enrichment analysis. In enrichment result of molecular function, Wnt protein binding ranked first in the high *ITGBL1* expression group, which proved the close association between *ITGBL1* expression and Wnt signaling pathway in the interstitial tissue of CRC. Based on the GSEA analysis of GSE39582, 31 genes with enrichment function in KEGG_WNT_SIGNALING_PATHWAY were obtained. Intersection was performed between those 31 genes and upregulated genes in the high *ITGBL1* expression group in CRC parenchymal tissue and interstitial tissue, respectively. In interstitial tissue, seven intersected genes (*SFRP2, WNT2, FZD1, FZD7, FZD8, SFRP4*, and *DKK2*) were attained. In parenchymal tissue, two intersected genes (*SFRP4* and *DKK2*) were obtained. So, *SFRP4* and *DKK2* were significantly expressed in both parenchymal tissue and interstitial tissue. The encoded proteins of those genes were mainly located in the cell membrane or ECM as the extracellular signal protein of Wnt signaling pathway. Co-expression analysis was performed between the above seven genes and *ITGBL1* using 222 microarray data of CRC obtained in The Cancer Genome Atlas (TCGA) (15) by using cBioPorta (16). It was observed that *SFRP4* was the most relevant gene with *ITGBL1* expression, and other genes were also correlated with *ITGBL1* in varying degrees (Figure 2). PrePPI was performed to analyze the interaction relationship between ITGBL1 and the seven proteins. Cytoscape (17) was applied to construct protein–protein interaction network by selecting proteins with combining probability more than 0.8. It is found that six out of seven of those genes (proteins) could be bound to ITGBL1 indirectly, and CTNNB1 (β-catenin) was the key protein (Figure 3). Hence, those six Wnt-related genes were upregulated in the high *ITGBL1* expression group and had co-expression relationship with *ITGBL1*. The encoded proteins of those six genes could bind to CTNNB1, and CTNNB1 could bind to ITGBL1, which suggested that CTNNB1 played an important role in the regulatory mechanism of ITGBL1 in the metastasis of CRC. According to the GSEA analysis of GSE39582, REACTOME_BETA_CATENIN_INDEPENDENT_WNT_ SIGNALING ranked first in the enrichment of REACTOME, which also suggested the important role of CTNNB1 in the relationship of ITGBL1 and Wnt signaling pathway.

## Discussion

ECM is closely related to the invasion and metastasis of tumors and involved in the formation of the tumor microenvironment. ITGBL1 is located in the ECM and related to the tumor microenvironment. This study analyzed the expression change of *ITGBL1* at different stages of CRC and determined that *ITGBL1* expression was associated with the metastasis of CRC. By analyzing the expression profile of CRC, it was discovered that *ITGBL1* was closely related to extracellular Wnt signals (*SFRP2, WNT2, FZD1, FZD7, FZD8, SFRP4*, and *DKK2*) *via CTNNB1*. CTNNB1 (β-catenin) is a key protein of Wnt signals and linked to the development of tumor (18). As reported, CTNNB1 could affect autophagy in glioblastoma, and cell autophagy was related to the tumor microenvironment (19) and was also involved in adrenocortical carcinomas (20). Molecular mechanism related in CRC was correlated with CTNNB1; for instance, genetic variations of the CTNNB1 were related to the progression of CRC (21). SNX3 could inhibit the metastasis of CRC via downregulating β-catenin (22), miR-150 could suppress the metastatization process of CRC by inhibiting β-catenin (23). FOXM1 could promote the growth and metastatization process of CRC by activating β-catenin (24). In the serum of patients with CRC, the expression of β-catenin was higher than that in normal people (25). In addition to CTNNB1, this study also found that extracellular Wnt signals (*SFRP2, WNT2, FZD1, FZD7, FZD8, SFRP4*, and *DKK2*) were correlated with *ITGBL1* expression. Those proteins and ITGBL1 were cell membrane or extracellular proteins and had higher relation with *ITGBL1* in the interstitial tissue of CRC. Those proteins were closely related to the development of CRC. For instance, methylation could inhibit gene expression and hypermethylation of *SERP2* and was negatively associated with the invasion of CRC (26). Depletion of WNT2 could inhibit CRC (27), while cancer-associated fibroblasts (CAFs)-derived WNT2 could promote the progression of CRC. Moreover, FZD8 might be a WNT2 receptor (28, 29), while miR-375 could suppress human CRC metastasis by inhibiting FZD8 (30). FZD1 is a Wnt responsive gene in colon-derived tissues which were expressed in CRC, and paracancerous normal mucosa was involved in Wnt signaling within the tumor microenvironment (31). RNA interference-mediated silencing of *FZD7* inhibited invasion in CRC, and its expression was associated with the activation of Wnt signaling (32). High expression of SFRP4 was correlated with advanced CRC (33), and CRC patients with overexpressed SFRP4 had lower overall survival (34). DKK2 expression accelerates aerobic glycolysis and promotes angiogenesis in CRC (35). Therefore, those extracellular Wnt signals were related to activation of Wnt signaling pathway and the metastatization of CRC. The study established that the regulatory mechanism of ITGBL1 in the development and metastatization of CRC might be closely related to those proteins. Hence, ITGBL1 is closely associated with the metastatization of CRC and involved in the tumor microenvironment.

## Data Availability Statement

All datasets generated for this study are included in the article/Supplementary Material.

## Author Contributions

LQ designed and performed the study, analyzed the data, and wrote the manuscript. FS collected the data and literature. YD revised and approved the final manuscript.

### Conflict of Interest

The authors declare that the research was conducted in the absence of any commercial or financial relationships that could be construed as a potential conflict of interest.

## Supplementary Material

Table S1 GSEA enrichment analysis of the high ITGBL1 expression group and the low ITGBL1 expression group. Screening results of ITGBL1-related differentially expressed genes (DEGs) in parenchyma and interstitial of CRC.
